# An International Comparative Study of Students' Scientific Explanation Based on Cognitive Diagnostic Assessment

**DOI:** 10.3389/fpsyg.2021.795497

**Published:** 2021-12-17

**Authors:** Tao Hu, Jing Yang, Rongxiu Wu, Xiaopeng Wu

**Affiliations:** ^1^College of Teacher Education, Faculty of Education, East China Normal University, Shanghai, China; ^2^School of Education, Indiana University, Bloomington, IN, United States; ^3^Neag School of Education, University of Connecticut, Mansfield, CT, United States; ^4^Faculty of Education, Shaanxi Normal University, Xian, China

**Keywords:** scientific explanation, cognitive diagnosis, TIMSS, international comparison, learning path

## Abstract

Scientific explanation is one of the most core concepts in science education, and its mastery level is crucial for a deep understanding of the nature of science. As a new generation of assessment theory, cognitive diagnostic assessment (CDA) can get the knowledge of students' mastery of fine-grained knowledge. Based on the extant research, this research has formed eight attributes of scientific explanation concepts. By coding the Trends in International Mathematics and Science Study (TIMSS) test items, a CAD tool was formed. Data collected from 574 Grade 4 students in Hangzhou, China, combined with the data of the United States, Singapore, Australia, the United Kingdom, and Russia, were used in our study. The Deterministic Inputs, Noisy “And” gate (DINA) model was used to analyze the results from three aspects: the probability of mastery of attributes, the international comparison of knowledge states, and the analysis of learning paths. This study provided a new perspective from a CDA approach on the assessment of scientific explanation.

## Introduction

One of the goals of science education is to help students become scientifically literate individuals capable of participating in science discourses and practices (McNeill et al., 2006; Anderson, 2013). To meet this goal, cultivating students to construct scientific explanations and supporting science teachers in assessing students' explanations is essential for science learning and teaching around the world (National Research Council, 1996; Ministry of Education, P. R. China, 2011; NGSS Lead States, 2013). For example, the Nest Generation Science Standards (NGSS) Lead States in 2013 include “constructing scientific explanation” as one of the eight science and engineering practices. The National Research Council (NRC) emphasizes that “scientists evaluate the explanations proposed by other scientists by examining evidence, comparing evidence, identifying faulty reasoning, pointing out statements that go beyond the evidence, and suggesting alternative explanations for the same observations” (p. 148). The Chinese Science Curriculum Standards (Ministry of Education, P. R. China 2011, p. 13–14) specifies that “students should be able to know scientific explanations that are based on empirical evidence, scientific knowledge, and scientific reasoning.”

Assessing students' ability to construct scientific explanations is crucial for science teaching and learning. Researchers designed tasks that allow students to provide written responses to an investigated question or a phenomenon [e.g., McNeill, 2011; Yao and Guo, 2018]. Researchers then evaluate students' written explanations with a predetermined rubric. Such assessments could provide both summative and formative feedback; however, it is challenging to offer timely feedback for a large group of students. A more effective assessment is needed to report students' ability to construct scientific explanations. This article assessed students' ability to construct scientific explanations with an analysis on the TIMSS 2011 data from a cognitive diagnostic assessment (CDA) approach.

## Review of Relevant Literature

This study was grounded in two areas of the literature: (1) the meaning of scientific explanation and how science educators assess the quality of students' scientific explanation traditionally and (2) the promise of cognitive diagnostic modeling (CDM) for assessing students' ability to construct scientific explanations *via* large-scale datasets.

### Scientific Explanation and Associated Assessments

What a scientific explanation constitutes is different for philosophers, research scientists, and science educators (Edgington, 1997). Philosophers concern the ideological and historical aspects of scientific explanation. Research scientists apply different explanation models in practices (Edgington, 1997; Alameh and Abd-El-Khalick, 2018). The nature of scientific explanations is a set of implicitly shared cultural elements within the specific field (Edgington, 1997). Scientific explanations in science education have looser philosophical grounds (Edgington, 1997) and seem to be left undefined among researchers and practitioners (Alameh and Abd-El-Khalick, 2018). Constructing scientific explanation in K-12 context serves at least two purposes: (1) as means to evoke students' conceptual understanding of scientific phenomena and mechanisms and (2) as guidance for engaging students in scientific inquiry (Kuhn and Reiser, 2005). Researchers who emphasize students' understanding of science concepts view scientific explanations as an application of theory, facts, or principles to make sense of a phenomenon (Tang, 2016; Yao and Guo, 2018). The phenomenon to be explained is derived from premises (e.g., laws, theories, or observables) and is generally in no doubt (Osborne and Patterson, 2011). For example, one of the scientific explanation tasks developed by Yao and Guo (2018) requires students to explain why the “red soup” side of a “Yuanyang hotpot” always boils first. Students apply physics concepts around heat transfer to explain the phenomenon. Drawing from philosophy of science, research in science education, and standard documents [e.g., NGSS, NRC, Ministry of Education, P. R. China, 2011], Yao and Guo (2018) develop a phenomenon-theory-data-reasoning (PEDR) framework to conceptualize scientific explanation in the K-12 setting.

Researchers and science educators who focus on engaging students in scientific practices use the notion of scientific explanation with the features of scientific argumentation (Kuhn and Reiser, 2005; McNeill et al., 2006; Berland and Reiser, 2009; Braaten and Windschitl, 2011). These researchers view the construction of scientific explanation as an inherent part of scientific inquiry that students develop evidence-based explanations through their investigations (Kuhn and Reiser, 2005; McNeill et al., 2006; Berland and Reiser, 2009; Ruiz-Primo et al., 2010). For example, McNeill et al. (2006) propose a claim-evidence-reasoning (CER) model of scientific explanation to help students “justify their claims using appropriate evidence and scientific principles” (p. 155). Usually, students are involved in complex inquiry tasks in which the claim to be made has less certainty. In addition, they often encounter contradictory evidence or data that require them to justify in what way the evidence results in a certain claim. Osborne and Patterson (2011) point out that these elements are features of an argument (Toulmin, 1958). Researchers such as McNeill argue that the complexity of such practices and the student difficulties demand the conflation of explanation and argumentation. Their scientific explanation of the CER model is also consistent with school cultures and the standard documents (McNeill et al., 2006).

In this study, we used the notion with an emphasis on student's conceptual understanding of scientific phenomenon and mechanism. Here, scientific explanation was used as an individual effort to make sense of a phenomenon by applying theory, facts, or principles to scientific data (Tang, 2016; Yao and Guo, 2018). Engaging in the construction of scientific explanations, students develop an understanding of science content knowledge, and also the nature of scientific knowledge (Sandoval, 2001; Ruiz-Primo et al., 2010). Current efforts for assessing student's scientific explanations are mostly qualitative by nature, relying on student's written scientific explanation as a product of a curriculum (e.g., McNeill, 2011; Yao and Guo, 2018). In these studies, researchers developed rubrics for assessing students' written explanations in response to an investigated question or a phenomenon.

### Assessments of Cognitive Diagnostic Models *via* Large-Scale Datasets

Assessments at a qualitative end provide both summative and formative feedback; however, it is challenging to offer timely feedback for a large group of students. A more effective assessment is needed to report students' ability to construct robust scientific explanations. Achievement-based assessments, such as the Program for International Student Assessment (PISA), Trends in International Mathematics and Science Studies (TIMSS), and National Assessment for Educational Progress (NAEP), have the potential to provide students with timely diagnostic feedback and enable large-scale assessments. Each of the three measures holds unique features in terms of their purpose, population, and content (McGrath, 2008). The purpose of NAEP is to establish benchmarks of the performance of students in the United States, whereas PISA and TIMSS are the two major international large-scale assessment programs that provide comparative information (McGrath, 2008; Breakspear, 2012; OECD, 2013a,b; Wu et al., 2020, 2021b). PISA emphasizes the yield of the education system and students' competencies of applying knowledge and skills in authentic contexts, whereas TIMSS and NAEP emphasize school-based curricular. In terms of population, PISA targets students aged 15, TIMSS targets students in Grades 4 and 8, and the target population of NAEP is students in Grades 4, 8, and 12. The age-based feature of PISA distinguished it from the grade-based feature of TIMSS and NAEP. The science content areas are organized differently in PISA, TIMSS, and NAEP. For example, physical science is included as one content area in NAEP but is split into physics and chemistry in TIMSS. In this study, we selected TIMSS due to the following two reasons: (1) the internationally comparative information provided by TIMSS serves for our research purpose, and (2) there exists successful use of applying CDM analysis on TIMSS data (Chen et al., 2017; Wu et al., 2020, 2021b).

However, TIMSS cannot be readily applied to assess students' ability to construct scientific explanations due to the following reasons. First, the IRT model (Lord and Novick, 2008) adopted by TIMSS conflates examinees' latent ability into a few dimensions (Yamaguchi and Okada, 2018), failing to report fine-grained skills (or attributes) needed to assess students' ability to construct a scientific explanation. Second, items in TIMSS were originally designed to assess a mixture of students' fundamental knowledge and critical competencies (Wu et al., 2020, 2021c), which places a challenge to isolate attributes for a scientific explanation from the raw data.

As a new generation of assessment theory, CDA makes substantial assumptions about the process and knowledge structure that learners use in completing tasks to guide diagnosis, with a combination of cognitive science and psychometrics. Cognitive diagnosis requires precise specifications to describe the item's characteristics that trigger the cognitive process (Embretson, 1998). It aims to provide formative diagnostic feedback through fine-grained reports on learners' skill mastery (Tatsuoka, 1983; Embretson, 1998; Hartz, 2002; DiBello et al., 2007). In the past 30 years, cognitive diagnosis has been developed, especially in the field of education and psychometrics. CDM is especially suitable for decomposing the multidimensional content in assessment tools to provide clear information about the subject, which can help experts to make an accurate diagnosis and guide their decision-making. From complex cognitive stimuli in educational psychometrics to responsive clinical assessment, appropriate cognitive diagnosis can accurately classify and ultimately accurately diagnose where and how the subject is defective (Templin and Henson, 2010). As traditional tests, cognitive diagnosis requires detailed empirical evidence and a theoretical basis to specify the basis of the item to support the inferences and interpretations drawn from the diagnostic assessment (Yang and Embretson, 2007). However, it is worth noting that the key elements of CDA are not unique to these models but derived from other major psychological measurement and statistical frameworks, such as classical testing theory (CTT, Thissen, 2001), item response theory (IRT, de Ayala, 2009), Bayesian estimation (Lynch, 2007), and so on. CDMs are a class of psychometric models that combine modern statistical methods with cognitive theories and therefore produce feedbacks that reflect the cognitive and psychological characteristics of the subjects (Templin and Henson, 2010; Wu et al., 2020, 2021c). It holds great promise in providing fine-grained feedback (Leighton and Gierl, 2007; Templin and Bradshaw, 2014; Chang et al., 2021). For diagnostic purposes, CDMs could identify multiple criterion-referenced interpretations for numerous attributes in solving the test items. Therefore, the associated feedback can help students and teachers to discover ones' strengths and weaknesses in a set of attributes (Rupp and Templin, 2008). There has been increasing interest in using CDMs for educational and psychological assessments recently due to its potential in integrating the test objective into the cognitive models (Stout, 2002; Tatsuoka, 2002; Chen and Chen, 2016).

In the field of mathematics education, a variety of CDMs have been fitted to the TIMSS assessment data to provide readily useful evidence for researchers and educators on fine-grained attributes (Greeno, 1991; Rumelhart, 1991; Schneider and Graham, 1992; Zhan et al., 2018; Carpenter and Moser, 2020). In science education, there are very few studies that fit CDMs to the TIMSS data for science learning and teaching assessment. Kim et al. (2015) extracted nine attributes from the TIMSS 2011 science data to discover the characteristics of Korean middle school students' science learning based on cognitive diagnostic theory. Among the nine attributes, Korean students considered “use models,” “interpret information,” “draw conclusions,” and “evaluate and justify” as easier attributes, and considered “recall or recognize,” “explain,” “classify,” “integrate,” and “hypothesize and design” as harder attributes. Zhan et al. (2019) applied a multiorder CDM on the science assessment data of PISA 2015 to assess scientific literacy. They treated scientific literacy as a third-order latent trait that consists of “competencies,” “knowledge,” “contexts,” and “attitudes.” Results highlighted that knowledge was the most influential attribute on scientific literacy. To our knowledge, CDMs for assessing students' ability to construct scientific explanations have yet to be developed and validated.

This study aimed to apply CDM to assess students' abilities and construct scientific explanations with TIMSS 2011 dataset. Through the analysis of cognitive attributes, cognitive diagnosis integrates the test objectives into the cognitive model. Cognitive diagnosis captures the students' cognitive process when items are answered. Thus, it reflects the subjects' internal knowledge acquisition and their mastery of fine-grained knowledge states. With this, we can understand their internal knowledge mastery states and obtain the participants' learning situation through the relationship between the knowledge chains to better guide learning. Wu et al. (2020, 2021c) put forward a method for constructing learning paths and learning progressions based on cognitive diagnosis theory, which provides a reference for further in-depth analysis of CDA results. Here, we used TIMSS 2011 Grade 4 science test items and selected data from the United States, Singapore, Russia, the United Kingdom, Australia, and also data collected from Hangzhou, China, for a comparative analysis of attribute mastery and tried to find out what problems might exist in terms of students' scientific interpretation. Eight attributes were extracted in this study based on the phenomenon-theory-data-reasoning (PTDR) framework developed by Yao and Guo (2018). We further compared the typical knowledge states among the different countries, that is, to analyze the ranking of the number of knowledge states and to obtain the characteristics of the scientific explanation for different countries. Based on this, the learning path of scientific explanation for students in Hangzhou, China, was further constructed. We tried to make predictive assumptions about students' learning and subsequently provided a reference for students' personalized learning arrangements.

## Methodology for Cognitive Model Construction

To assess students' ability to construct scientific explanations, we started with the construction of a cognitive model for the cognitive diagnostic test. By fitting an appropriate CDM to the TIMSS 2011 data, we can obtain the knowledge states for each student. According to the theory of cognitive diagnosis, students' knowledge states can be reflected by cognitive attributes; the attributes are connected to test items *via* Q-matrix. This section first introduced how we constructed attributes for assessing students' ability to construct scientific explanations and then described the associated Q-matrix.

### Cognitive Attributes for Scientific Explanation

Attributes are at the heart of cognitive attributes as their quality directly determines the effectiveness of the CDA (Wu et al., 2020, 2021a). Cognitive attributes took multiple meanings in the field of measurement ranging from knowledge and thinking skills needed to solve a test item (Tatsuoka et al., 2004; Dogan and Tatsuoka, 2008), to process skills and knowledge structures needed to complete a task (Leighton and Gierl, 2007). In this study, we defined cognitive attributes for constructing scientific explanation as a set of thinking skills and constructed eight attributes (Table 1) based on the existing framework of scientific explanation.

**Table 1:** Definitions of the cognitive attributes for assessing student's ability to construct scientific explanation.

**Code**	**Attributes**	**Definition**
OP	Observing the phenomenon	Must observe the pictures of scientific phenomena when answering the test questions (only observing the pictures can make the correct answer)
DP	Describing phenomena	When solving the problem, it is necessary to describe the scientific phenomenon and make the phenomenon in the problem specific. For instance, “nocturnal animals are more active,” you need to restore “active” to specific behaviors such as “frequently running” and “howling.”
OD	Obtaining data	Use the information about the phenomenon clearly provided in the title, such as text description of the phenomenon, data chart information (if the information in the question is not needed, it will be scored 0 if you can directly answer with the original concept understanding)
AD	Analyzing data	Analyze and process data that cannot be directly concluded. The data presented in the question cannot be directly used to draw conclusions. It needs to be analyzed and processed to become evidence before conclusions can be drawn (if you have given relevant data, you cannot draw a conclusion based on the data immediately, you need to process the data first).
UF	Using facts	Use scattered knowledge or facts. Students can describe and “explain” daily phenomena based on their daily experiences, or scattered facts learned from books, the Internet, and other media
CR	Constructing reflection	Connect the phenomenon in the question with the concept used to answer the question. Establish a mapping relationship between scientific concepts and phenomena (such as linking “insects” with the characteristics of insects such as six legs, two pairs of antennae, etc.). Use key variables as clues to choose (direct autonomous selection) scientific concepts, laws, principles or theories (at the mapping level)
ST	Systematic use of theory	Use two or more concepts, theories or principles involved in the phenomenon in the title to analyze. Use two or more scientific concepts, laws, principles or theories to conduct system thinking
SR	Scientific reasoning	Use the information in the topic to perform scientific reasoning activities such as induction, deduction, and analogy.

As reviewed earlier, various frameworks were proposed for assessing students' ability to construct scientific explanations with different instructional goals. For example, the CER framework (McNeill et al., 2006) was proposed to engage students in constructing scientific explanations toward their own investigation, whereas the PRO (Tang, 2016) and PTDR (Yao and Guo, 2018) frameworks emphasized more on the application of scientific knowledge to explain phenomena. The purpose of this study was to assess students' ability in explaining science phenomena, so we extended the PTDR framework (Yao and Guo, 2018) to construct the attributes. The PTDR framework more clearly explains the process and attributes of scientific explanation, which is more suitable for cognitive diagnosis. Based on the PTDR framework and the TIMSS 2011 assessment framework, eight attributes were constructed along with their corresponding definitions (Table 1). These are the following: observing phenomenon (OP), describing phenomena (DP), obtaining data (OD), analyzing data (AD), using facts (UF), constructing reflection (CR), systematic use of theory (ST), and scientific reasoning (SR).

### Q-Matrix

The eight attributes for assessing students' ability to construct scientific explanations above were connected to the test items in TIMSS 2011 *via* a Q-matrix. We selected 30 TIMSS test items that were jointly tested by the students in the six countries we studied, which resulted in a 30 × 8 matrix. A value of 1 for the Q-matrix entry indicates that an attribute is measured for a corresponding item while 0 is not. According to the definition of the attributes of scientific explanation in Table 1, two groups of experts coded the test items without a mutual exchange of info and finally formed the Q-matrix of the test, as shown in Table 2.

**Table 2:** Q-matrix of test items in TIMSS.

	**OP**	**DP**	**OD**	**AD**	**UF**	**CR**	**ST**	**SR**
Item 1	0	0	1	0	1	1	0	0
Item 2	0	0	1	0	1	0	0	0
Item 3	1	1	1	1	1	1	0	0
Item 4	1	1	1	1	1	1	0	0
Item 5	1	1	1	1	1	1	0	0
Item 6	1	1	1	1	1	1	0	0
Item 7	1	1	1	1	1	1	0	0
Item 8	0	1	0	0	1	0	0	0
Item 9	0	0	1	0	1	0	0	0
Item 10	0	1	0	0	1	1	0	0
Item 11	1	0	1	1	1	1	1	0
Item 12	1	1	1	1	1	1	1	0
Item 13	1	0	1	1	1	1	1	1
Item 14	1	0	1	1	1	1	1	1
Item 15	0	0	0	0	1	0	0	0
Item 16	0	0	1	1	1	0	0	0
Item 17	0	0	0	0	1	1	0	0
Item 18	0	0	0	0	1	0	0	0
Item 19	1	0	1	0	1	1	1	0
Item 20	1	0	1	0	1	1	0	0
Item 21	0	0	0	0	1	1	0	0
Item 22	0	0	0	0	1	1	1	1
Item 23	0	0	0	0	1	1	0	0
Item 24	0	1	0	0	1	1	1	0
Item 25	0	1	0	0	1	1	1	0
Item 26	1	0	1	1	1	1	1	1
Item 27	0	0	0	0	1	0	0	0
Item 28	1	0	1	1	1	1	1	0
Item 29	1	0	1	1	1	0	0	0
Item 30	1	0	1	1	1	0	0	0

It can be seen from Table 2 that the eight attributes of scientific explanation all have at least one item to test, which deems reasonable in the distribution of attributes and can provide more diagnostic information for model diagnosis.

### Testing of Tools for CDA

This study selected 574 Grade four students from two schools in Hangzhou, China (CHZ) for the test. The time length for this test was 90 min. A uniform scoring standard was used for each test, and it was strictly consistent with the TIMSS test scoring standard. After calculation, the reliability of the test α = 0.795, which had a high degree of credibility. Among the model selection, this study selected the commonly used DINA model. This model assumes that the participant must master all the attributes of the item to complete a certain item. The absence of any attribute will make the probability of correctly answering the item very low. It belongs to a completely uncompensated cognitive diagnosis model, which has been widely used in the practice of educational assessment. To further test the quality of the test questions, we also tested the fit and discrimination of the items separately.

### Item Level Fit

The degree of fit between the test item and the model is significant in CDA. To a certain extent, it explains the quality of the cognitive diagnostic test item. In this study, the root mean square error of approximation (RMSEA) was used as the test parameter for item fit. The RMSEA of the 30 items was shown in Table 3.

**Table 3:** RMSEA parameters of 30 test items for scientific explanation.

**Item 1**	**Item 2**	**Item 3**	**Item 4**	**Item 5**	**Item 6**	**Item 7**	**Item 8**	**Item 9**	**Item 10**
0.0227	0.0494	0.0070	0.0366	0.0443	0.0420	0.0693	0.0508	0.0096	0.0663
**Item 11**	**Item 12**	**Item 13**	**Item 14**	**Item 15**	**Item 16**	**Item 17**	**Item 18**	**Item 19**	**Item 20**
0.1420	0.0747	0.1122	0.0167	0.0039	0.0169	0.0333	0.0144	0.0078	0.0417
**Item 21**	**Item 22**	**Item 23**	**Item 24**	**Item 25**	**Item 26**	**Item 27**	**Item 28**	**Item 29**	**Item 30**
0.1019	0.0417	0.0365	0.0179	0.0561	0.0343	0.0465	0.0677	0.0418	0.0919

According to the standard of RMSEA, the closer the value of RMSEA is to 0, the smaller the deviation of the fit and the better the fit effect. Oliveri and von Davier (2011) take 0.1 as the critical value of item fit, that is, RMSEA > 0.1 indicates that the item fit is poor. According to this standard, it can be seen in Table 3 that, except for Item 11, Item 13, and Item 21, the test item parameter values were slightly higher, whereas the other parameters were all <0.1. It showed that the item level of fit was acceptable and reasonable.

### Item Differentiation

The degree of discrimination of test items is an important indicator in evaluating the quality of a test. The discrimination of the cognitive diagnostic test is defined as


$$\begin{array}{l} d_{j}=P_{j}\left(1\right)-P_{j}\left(0\right) \end{array}$$

Among them,*P*_*j*_(1) refers to the probability of answering the item correctly with grasping all the attributes of item j; *d*_*j*_(0) refers to the probability of answering the item correctly without grasping all the attributes of item j. The smaller the *d*_*j*_ , the smaller the influence of whether mastering the attribute on correctly answering this item, that is, the smaller the degree of discrimination. On the contrary, the greater the degree of discrimination. A large discrimination is a sign of high-quality test items. Through applying the GDINA package in the R package, the distinction between different items was shown in Table 4.

**Table 4:** Discrimination statistics of 30 test items for scientific explanation.

**Item 1**	**Item 2**	**Item 3**	**Item 4**	**Item 5**	**Item 6**	**Item 7**	**Item 8**	**Item 9**	**Item 10**
0.5332	0.3452	0.2671	0.4274	0.6411	0.0474	0.8726	0.2301	0.3484	0.0180
**Item 11**	**Item 12**	**Item 13**	**Item 14**	**Item 15**	**Item 16**	**Item 17**	**Item 18**	**Item 19**	**Item 20**
0.1653	0.2379	0.2974	0.2861	0.1696	0.2232	0.1299	0.3547	0.2195	0.4276
**Item 21**	**Item 22**	**Item 23**	**Item 24**	**Item 25**	**Item 26**	**Item 27**	**Item 28**	**Item 29**	**Item 30**
0.2492	0.5855	0.4853	0.2099	0.2823	0.3369	0.0242	0.3629	0.5997	0.5931

From Table 4, most of the test items had a high degree of discrimination, and a small number of items had a low degree of discrimination, especially the discrimination degree of Item 6 and Item 10 was <0.1. The quality of these two test items was relatively low; however, considering the fit effect of all the test, these items were still retained.

Based on the above analysis, the results of RMSEA showed that 30 items have a good fit with the DINA model, and the test results of item discrimination showed that 30 items had good discrimination, and the test had a good degree of discrimination (α = 0.795). Therefore, the 30 items selected here are appropriate for performing cognitive diagnostic analysis on the eight attributes for scientific explanation. The following section presents our comparative analysis and results of attribute mastery, knowledge states ranking, and the learning path.

## Cognitive Diagnostic Analysis and Results

Cognitive diagnostic assessment can provide each student the mastery of different attributes; that is, the knowledge states of each student will be obtained. This study selected five countries with representative TIMSS scientific test results from the United States (USA), Russia (RUS), Australia (AUS), Singapore (SGP), and the United Kingdom (ENG) and compared the results with that collected from Hangzhou, China. In the selection process of data, we first considered the distribution of the data and selected representative countries from different states. Considering the selection of influential countries in the TIMSS test as the comparison object, three aspects were compared overall: the international comparative analysis of attribute mastery, knowledge states ranking, and the learning path analysis of scientific explanation.

### International Comparative Analysis of Attribute Mastery

The study assessed the model with the commonly used cognitive diagnosis model, DINA model and obtained the mastery of the attributes of each student in each country with the DINA package in R. Overall, the mastering probabilities of the eight attributes in each country were shown in Table 5.

**Table 5:** Results of the eight attributes mastered in six countries for scientific explanation.

	**OP**	**DP**	**OD**	**AD**	**UF**	**CR**	**ST**	**SR**
CHZ	50.17%	89.37%	55.23%	54.88%	56.97%	96.86%	55.23%	59.23%
USA	71.85%	68.49%	100.00%	73.53%	68.49%	95.80%	62.18%	60.92%
SGP	100.00%	68.55%	70.72%	62.91%	61.17%	100.00%	97.40%	67.68%
RUS	71.70%	85.85%	84.28%	65.41%	71.70%	92.77%	87.74%	67.61%
ENG	74.22%	62.39%	74.22%	80.80%	75.56%	95.20%	55.13%	61.05%
AUS	74.37%	65.22%	88.79%	83.07%	78.49%	98.63%	64.41%	70.48%

**Figure 1:**
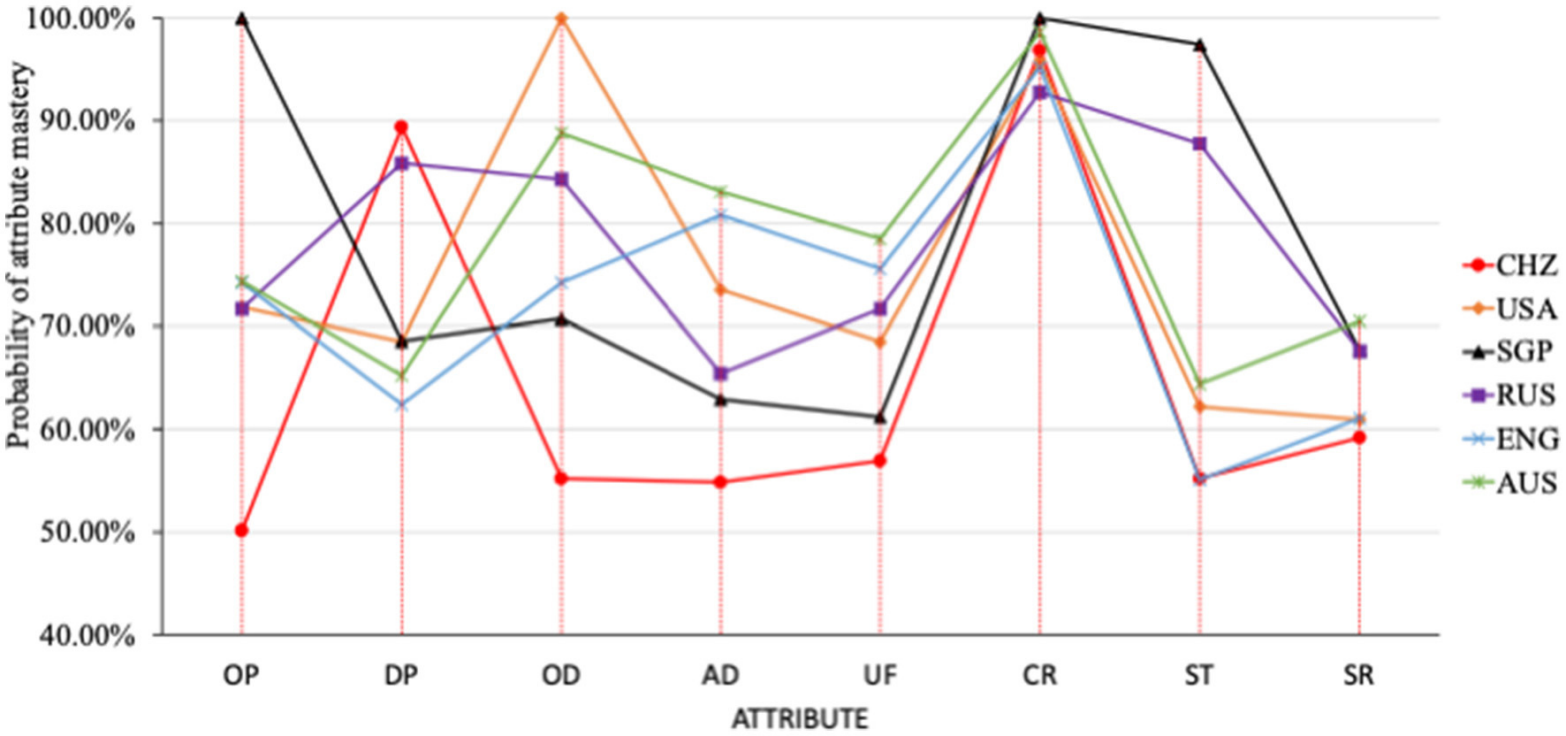
The probability of students' mastery of each attribute of scientific interpretation in different countries.

According to Table 5, a line chart of the attributes of different countries was also shown in Figure 1.

According to Figure 1, the probability of mastery of attribute constructing reflection (CR) mastery was consistently high for all six countries, reaching more than 95%. Mastery probability for the rest attributes was different, resulting in different patterns for the six countries. CHZ students have a higher probability of attributes mastery for DP and CR. Their probability of attributes mastery for OP, OD, AD, and UF, ST, and SR were relatively lower (<60%). USA students had a higher probability of attributes mastery for OD and CR (>95%). The mastery probability of the rest four attributes is between 60 and 73%. SGP students had obvious advantages in the three attributes OP, CR, and ST, all of which were highest among the six countries. It also fully embodied the advantages of SGP in scientific explanation. RUS, ENG, and AUS students did not show apparent advantages or disadvantages of any attributes. Their mastery probability of each attribute was at an average level among six countries. It should be noticed that ENG and AUS demonstrated similar attribute mastery patterns.

### International Comparative Analysis of the Ranking of Knowledge States

The essence of the process of cognitive diagnosis is also the process of diagnostic classification. Therefore, CDM is also called the diagnostic classify model. The results of cognitive diagnosis could be accurately classified for the test participants to achieve the effect of instructing the students in accordance with their aptitude. In this study, the knowledge states of different countries were integrated with the same knowledge states being put together, and then, the top five knowledge states of different countries were concluded, which was shown in Table 6.

**Table 6:** The top five knowledge states in different countries.

	**1st**	**2rd**	**3rd**	**4th**	**5th**
CHZ	(1111 1111)	(0100 0100)	(1011 1111)	(0011 1111)	(0000 1000)
	44.25%	37.80%	4.18%	1.39%	1.39%
USA	(1011 1111)	(0110 0100)	(1111 1111)	(1111 1101)	(1111 1100)
	28.99%	26.47%	21.85%	5.46%	3.36%
SGP	(1011 1111)	(1100 0110)	(1111 1111)	(1111 0111)	(1110 0111)
	31.02%	27.33%	26.46%	4.99%	3.90%
RUS	(1111 1111)	(0100 0110)	(1011 1111)	(1111 1011)	(1110 1111)
	38.68%	13.84%	13.21%	6.60%	5.35%
ENG	(1011 1111)	(1111 1111)	(0100 0100)	(1111 1101)	(1111 1100)
	29.02%	19.20%	16.85%	8.48%	8.48%
AUS	(1111 1111)	(1011 1111)	(1111 1101)	(0100 0100)	(1111 1100)
	25.17%	19.45%	13.55	11.21%	7.78%

According to Table 6, students' knowledge states in CHZ, RUS, and AUS (1111 1111) ranked first, especially the proportion of knowledge states in CHZ accounted for 44.25%. It showed that a considerable number of students have mastered all the attributes of scientific explanation. To some extent, it indicated that most students of CHZ have a good grasp of scientific explanation. The USA's student knowledge states (1111 1111) ranked the third, which showed that most students in the USA did not master at least one of the attributes, and the cultivation of students' scientific explanation still needs to be worked on according to the need of different students. Additionally, almost all the knowledge states of CHZ students were in the top two, which accounted for 80% of the population. The distribution of other countries was relatively scattered; especially in USA, ENG, and AUS, the top four attributes all accounted for a larger proportion. It explained to a certain extent the polarization of CHZ, because 37.80% of the knowledge state (0100 0100) only mastered two attributes, whereas the distribution of other countries was comparatively more diverse.

### Analysis of the Learning Path of Scientific Explanation

**Figure 2:**
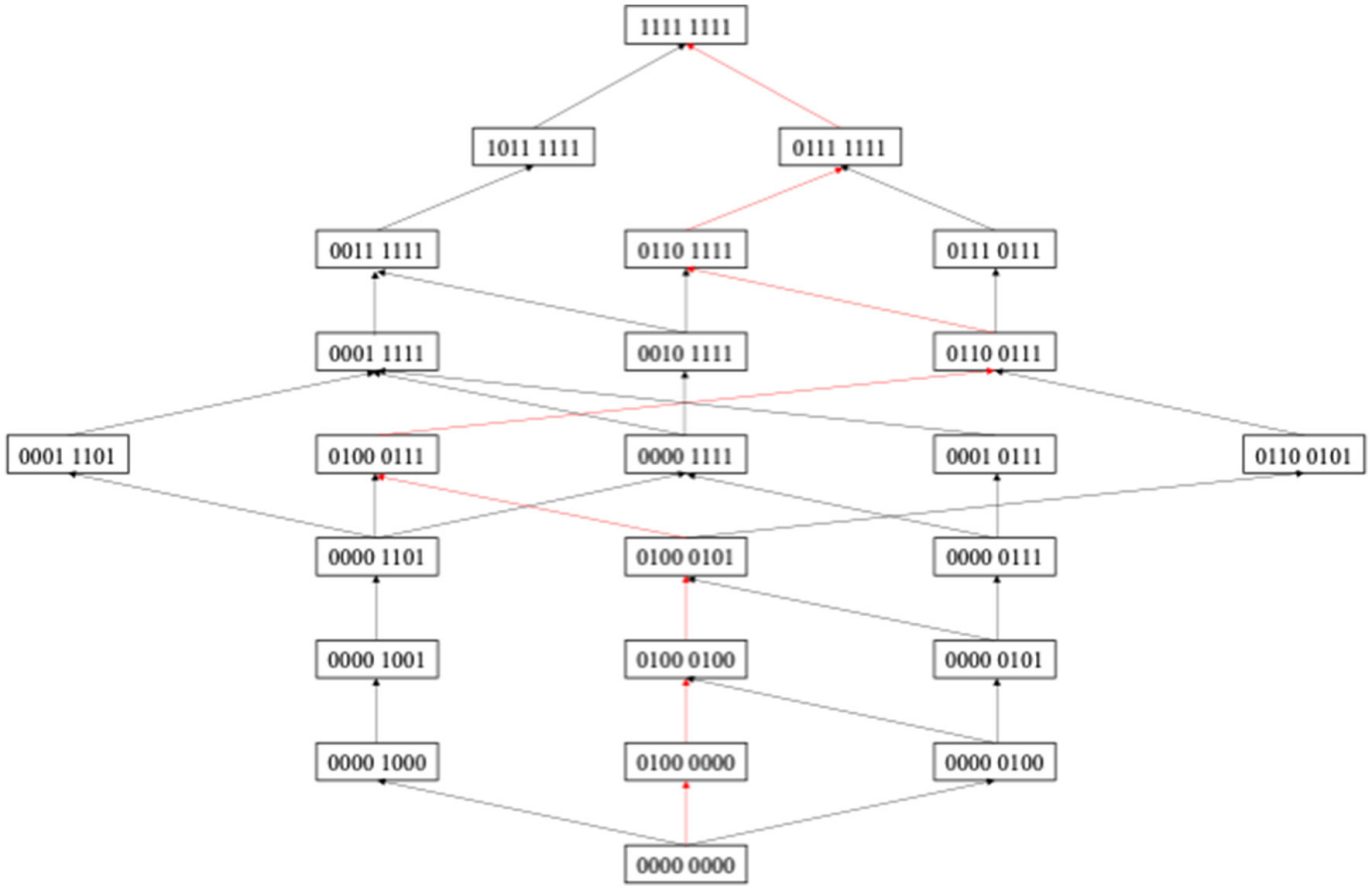
The learning path of CHZ students' scientific explanation.

Based on CDA, Wu et al. (2020) proposed a hypothetical construction method for learning path, which was based on the following two hypotheses. First, it is supposed that students acquire attributes step by step and attributes are to be mastered one by one; second, it is believed that the state of knowledge displayed by different students in a group is impacted by the inherent characteristics of the group, such as the teachers' teaching style, the students' learning resources, and so on. Therefore, the types of knowledge states shown by students in different levels can be regarded as a stage of student learning. According to this hypothesis, the changes in the group's knowledge states can reflect the learning path of this group to a large extent. Based on this method, this study constructed a learning path diagram for scientific explanation in China, which was shown in Figure 2.

According to Figure 2, the learning path reflected the inclusion relationship among different knowledge states, and it also reflected the knowledge state path from no attribute mastered (0000 0000) to finally all attributes mastered (1111 1111). From Figure 2, the learning paths of students showed diversity; that is, students can choose different paths to finally master all attributes. The red path in the figure represented the main learning path, because this learning path carried the largest number of people. It showed that this group of students was more inclined to this learning path during the learning process. Therefore, students also try to give priority to the main learning path, which is more in line with group learning resources, learning styles, learning habits, etc., and is also more in line with students' cognitive development rules, and learning may be more efficient. This learning path (0000 0000) (0100 0000) (0100 0100) (0100 0101) (0100 0111) (0110 0111) (0110 1111) (0111 1111) (1111 1111). The order of learning represented by this learning path was as follows: DP, CR, SR, ST, OD, UF, AD, and OP. This order may be inconsistent with the logical order of the subjects, but it embodies the “voice” of the students in the learning arrangement.

## Discussion and Implications

In this study, we introduced a CDM-based methodology to assess students' ability to construct scientific explanations using the TIMSS 2011 Grade 4 science dataset of the United States, Singapore, Russian, the United Kingdom, Australia, and data we collected from Hangzhou, China. CDM approach was selected due to its ability to provide readily useful evidence to researchers and educators on students' fine-grained attributes (Greeno, 1991; Rumelhart, 1991; Schneider and Graham, 1992; Zhan et al., 2018; Carpenter and Moser, 2020). Eight cognitive attributes of students' ability were employed to construct scientific explanations, and the attribute mastering patterns of different students were obtained, based on which we built a learning path for students' knowledge states of mastery. Results from our study agreed with previous studies about scientific explanation and scientific literacy at a coarse-grained level (McNeill et al., 2006; Yao and Guo, 2018; Zhan et al., 2019); the information provided at fine-grained levels further illustrated the relationship among the eight attributes.

We found that students' ability to construct scientific explanations was topic-specific, highlighting the relationship between students' understanding of content knowledge and their ability to construct scientific explanations. For example, we found students from Hangzhou, China, mastered higher-level attributes (e.g., “CR” and “ST”) on topics that they have a deeper understanding of the associated content knowledge (e.g., energy, force, and motion). However, they failed to master attributes at lower levels (e.g., “UF”) when their encountered unfamiliar topics. “UF” was considered as a basic level attribute in traditional assessment, whereas “CR” and “ST” were in-depth ones (Yao and Guo, 2018). A possible explanation was the TIMSS we use that involves knowledge in various fields. Still, the more in-depth investigations are often the more classic fields of science (such as force and magnetism). The knowledge of these traditional fields involved in the teaching materials is relatively solid. Still, the knowledge involved in the teaching materials is relatively narrow compared with other countries (such as the United States). Therefore, even if some test questions only require students to use basic scientific facts, they have hardly been exposed to this field, so they cannot complete the explanation of these projects. Thus, some students may have a poor grasp of the broad attributes of “UF” but have a better grasp of the attributes of “CRs” and “ST.” Our finding agreed with previous studies in science education. For example, Yao et al. (2016) showed that students' competencies in scientific explanations were advanced when the instruction of scientific explanation was integrated with the disciplinary core idea of energy. Applying a CDM analysis on the PISA2015 science test dataset, Zhan et al. (2019) concluded that the content knowledge students have mastered had the greatest influence on their scientific literacy. This finding implies that students' ability to construct scientific explanations appears to be domain-general knowledge, but we cannot assume such ability mastered from one content area is readily transferred into another. It is also necessary to include topic-specific scaffolds within a curriculum to improve students' ability to construct scientific explanations.

We demonstrated that the CDM is a promising tool to provide timely diagnostic feedback on students' mastery of attributes in scientific explanation, which was an advantage over traditional assessments based on a qualitative approach [e.g., McNeill et al., 2006; Yao and Guo, 2018]. Researchers such as McNeill et al. (2006) and Yao and Guo (2018) categorized students' scientific explanations into different levels and evaluated their scientific explanations as a summative report. Their studies successfully reflected the overall quality of individual students' scientific explanation, but they did not place their focus on students' mastery of individual attributes. In addition, the development of students' mastery of attributes is an evolving process, which placed challenges for qualitative approaches to provide timely feedback. Using a CDM approach, teachers could diagnose students' mastery of individual attributes. With this, they could select instructional materials, strategies, activities, and evaluation methods that emphasize the development of specific attributes accordingly.

Assessing students' ability using large-scale datasets enabled by CDM made it possible to reflect different attributes mastery patterns for students from different countries. The patterns allow us to delineate which attributes were considered as challenging for a certain group of students. For example, our study reported the different mastery levels on eight attributes of students from different countries. We found 44.25% of students from Hangzhou, China, mastered all eight attributes, which was the highest among the six countries. However, the rest of the students from China mastered a significantly fewer number of attributes. Comparing it to those in other countries, although fewer students mastered all eight attributes, the number of attributes mastered by individual students was higher. There appeared to be polarization in students' ability to construct scientific explanations in Hangzhou, China. Attribute mastery patterns reflect the uniqueness of learning resources and learning environments in different locations. CDM results provide an entry for educators to reflect their education system, standards, curriculum, and assessments and consider the cultural influences.

To our knowledge, using CDM to delineate the learning path for students' scientific explanations based on large-scale datasets is a novel approach in the field of science education. The learning path we constructed here was based on the arrangement of individual and group attribute mastery levels. Extracted from assessment-based data, our learning path was objective and scientific. The learning path represents the cognitive order that a specific group of students followed in a specific context, as opposed to the logical order of a discipline offered in the form of a curriculum. Such a learning path reflects students' learning process and promotes their development (Confrey et al., 2009), as opposed to reflecting a discipline-centered learning path. This sets the boundary between learner logic and subject logic (Corcoran et al., 2009). Combining results from individual students and their learning mode, the learning path that constructed *via* a CDM approach has the potential to provide guidance for science teachers to select instructional materials, strategies, activities, and evaluation methods. It should be emphasized that we distinguish between the learning path we constructed in this study and the well-studied “learning progression” [e.g., Songer et al., 2009] in the field of science education. Our learning path represents the order in which students master different attributes. Although our ultimate goal was the same as researchers studying learning progression (i.e., capturing the pathway how students master core concepts of scientific explanation), further evidence is needed to show that our learning pathway agrees with existing research on learning progression in science education. However, we demonstrated the potential of CDM and called upon the science educators and researchers to consider the novel tool for assessing students' ability to construct scientific explanations.

## Research Limitations

In this study, we adopted a CDM approach to mine science dataset in TIMSS 2011, extracted the mastery level of students' ability to construct scientific explanations, and constructed the learning path that reflects on students' learning progress. Our study provided a new perspective on assessing students' ability to construct scientific explanations by taking advantage of the existing large-scale dataset and possibilities to offer timely diagnostic feedback. However, there existed limitations in our study and room for improvement. First, the eight attributes we constructed for this study were extended from Yao and Guo (2018) PTDR framework, which was conceptualized by synthesizing research in the philosophy of science, science education, and standard document. Further research is to be conducted to validate whether these attributes truly capture students' knowledge and skills. Second, the eight attributes we constructed focused more on students' skills to construct scientific explanations, weighing less on the students' knowledge about scientific explanation (e.g., what constitute a scientific explanation). Third, the test items in TIMSS were not designed for assessing students' ability to construct scientific explanations. Although our results were in agreement with prior studies in science education at a coarse-grained level, further evidence should be collected to ensure the validity of using CDM as a diagnostic tool for scientific explanations on TIMSS data. Fourth, the learning path we constructed in this study was based on a cross-sectional dataset. The underlying assumption was that knowledge states captured from different students in a group at one time reflect different stages of individual student's learning, that is, the embodiment of group consciousness in the individual (Xin et al., 2015). This assumption is rational because collective characteristics of students' knowledge states are determined by the inherent factors within their group, such as learning resources, learning environment, and teaching strategies. Although this assumption is yet to be confirmed by empirical research, there exists successful use of cross-sectional dataset to reflect characteristics of longitudinal dataset *via* a CDM approach in mathematics education (Chen et al., 2017; Wu et al., 2020, 2021b). For future study, we are planning to take account of the analysis of longitudinal data to establish the reliability and stability of the method for science education.

## Data Availability Statement

The original contributions presented in the study are included in the article/Supplementary Material, further inquiries can be directed to the corresponding author/s.

## Ethics Statement

Ethical review and approval were not required for the study on human participants in accordance with the local legislation and institutional requirements. Written informed consent from the participants' legal guardian or next of kin was not required to participate in this study in accordance with the national legislation and the institutional requirements.

## Author Contributions

TH devised the conceptual framework, data interpretation, and manuscript writing. JY assisted with the development of conceptual framework, data interpretation, and manuscript construction. XW designed and directed the study, derived the models, and analyzed the data. RW assisted model construction and interpretation. Each of us contributed part of the manuscript writing. All authors contributed to the article and approved the submitted version.

## Funding

This work was supported by 2021 Humanities and Social Science Research project of Ministry of Education of China: Cognitive Diagnosis and Evaluation of Mathematics Core Literacy (21YJC880102) and 2020 Academic Innovation Ability Enhancement Plan for outstanding doctoral Students of East China Normal University (No. YBNLTS2020-003).

## Conflict of Interest

The authors declare that the research was conducted in the absence of any commercial or financial relationships that could be construed as a potential conflict of interest.

## Publisher's Note

All claims expressed in this article are solely those of the authors and do not necessarily represent those of their affiliated organizations, or those of the publisher, the editors and the reviewers. Any product that may be evaluated in this article, or claim that may be made by its manufacturer, is not guaranteed or endorsed by the publisher.
